# Trajectories of Adolescents Treated with Gonadotropin-Releasing Hormone Analogues for Gender Dysphoria

**DOI:** 10.1007/s10508-020-01660-8

**Published:** 2020-03-09

**Authors:** Tessa Brik, Lieke J. J. J. Vrouenraets, Martine C. de Vries, Sabine E. Hannema

**Affiliations:** 1 Department of Pediatrics, Leiden University Medical Centre, P.O. Box 9600, 2300 RC Leiden, The Netherlands; 2 Department of Pediatric and Adolescent Psychiatry, Curium-Leiden University Medical Centre, Leiden, The Netherlands; 3 Department of Medical Ethics and Health Law, Leiden University Medical Centre, Leiden, The Netherlands; 4 Department of Paediatric Endocrinology, Sophia Children’s Hospital, Erasmus Medical Centre, Rotterdam, The Netherlands

**Keywords:** Gender dysphoria, Transgender, Gonadotropin-releasing hormone analogues, Hormone treatment, Gender identity

## Abstract

Gonadotropin-releasing hormone analogues (GnRHa) are recommended as initial treatment for adolescents diagnosed with gender dysphoria, providing time to follow gender identity development and consider further treatment wishes without distress caused by unwanted pubertal changes. This has been described as an extended diagnostic phase. However, there are also concerns about the physical, neurocognitive, and psychosocial effects of this treatment. In this retrospective study, we document trajectories after the initiation of GnRHa and explore reasons for extended use and discontinuation of GnRHa. Treatment was considered appropriate in 143 (67%) of the 214 adolescents eligible for GnRHa treatment by virtue of their age/pubertal status, and all started GnRHa (38 transgirls, 105 transboys; median age, 15.0 years [range, 11.1–18.6] and 16.1 years [range, 10.1–17.9]). After a median duration of 0.8 years (0.3–3.8) on GnRHa, 125 (87%) started gender-affirming hormones (GAH). Nine (6%) discontinued GnRHa, five of whom no longer wished gender-affirming treatment. Thirteen had used GnRHa for longer than required by protocol for reasons other than logistics and regularly met with a mental health professional during this time, supporting the use of GnRHa treatment as an extended diagnostic phase. In conclusion, the vast majority who started GnRHa proceeded to GAH, possibly due to eligibility criteria that select those highly likely to pursue further gender-affirming treatment. Due to the observational character of the study, it is not possible to say if GnRHa treatment itself influenced the outcome. Few individuals discontinued GnRHa, and only 3.5% no longer wished gender-affirming treatment.

## Introduction

Increasing numbers of young people diagnosed with gender dysphoria are seen by pediatric endocrinologists. Gender dysphoria is the persistent feeling of incongruence between gender identity (sense of being a man, woman, or other) and the sex assigned at birth. The diagnosis gender dysphoria can be made if the *Diagnostic and Statistical Manual of Mental Disorders* (DSM-5) criteria are met (American Psychiatric Association, 2013). The prevalence of gender dysphoria among Dutch adolescents aged 12–18 years was recently estimated to be 1 in 6300 based on numbers of adolescents seeking medical treatment, with a ratio of transboys (assigned female at birth) to transgirls (assigned male at birth) of 1.9:1 (Wiepjes et al., 2018). Genetic, hormonal, psychological, and social factors may play a role, but the exact etiology of gender dysphoria remains unknown (de Vries & Cohen-Kettenis, 2012; Hembree et al., 2017; Martinerie et al., 2018).

Gender dysphoria in prepubertal children can be expressed by dislike of their physical sex characteristics and gender incongruent behavior. In many children, gender dysphoria will not persist, but if the gender dysphoric feelings intensify during puberty, they are thought to be unlikely to subside (de Vries & Cohen-Kettenis, 2012; Hembree et al., 2017; Zucker et al., 2011). When puberty starts (Tanner genital/breast stage 2) and gender dysphoria persists, adolescents are eligible to start with puberty suppression using gonadotropin-releasing hormone analogues (GnRHa) (Coleman et al., 2011; Hembree et al., 2017). GnRHa treatment aims to give the adolescent the opportunity to explore their gender identity and time to consider if they wish to pursue gender-affirming treatment while development of unwanted secondary sex characteristics is suppressed in order to reduce distress (Hembree et al., 2017; Zucker et al., 2011). Effects of GnRHa on pubertal development are reversible. This is in contrast to gender-affirming hormones which have largely irreversible effects on secondary sex characteristics and may compromise fertility after prolonged use (De Roo, Tilleman, T’Sjoen, & De Sutter, 2016; Hembree et al., 2017).

Short-term adverse effects of GnRHa are hot flushes at the start of the treatment and sometimes mood alterations and fatigue (Delemarre-van de Waal & Cohen-Kettenis, 2006; Hembree et al., 2017; Schagen, Cohen-Kettenis, Delemarre-van de Waal, & Hannema, 2016). Few data are available on long-term adverse effects. Bone mineral density may be affected (Klink, Caris, Heijboer, van, & Rotteveel, 2015; Vlot et al., 2017), and since puberty is an important period for brain development (Sisk & Zehr, 2005), puberty suppression with GnRHa might also influence brain development. There is a lack of studies investigating effects of GnRHa on the brain. One study examined executive function and concluded that GnRHa treatment had no detrimental effects on performance (Staphorsius et al., 2015). However, a longitudinal study among 25 adopted girls treated with GnRHa for early puberty reported a decrease in IQ from 100.2 ± 12.7 to 93.1 ± 10.5 with a significant decline of performance score during treatment, but it was concluded that the decrease in IQ was not clinically relevant (Mul et al., 2001). A limitation of the study was the lack of a control group. A second small cross-sectional study of girls treated with GnRHa because of precocious puberty found no significant difference in cognitive functioning, behavioral, and social problems compared to healthy age-matched controls, but the study did not have enough power to detect differences smaller than one standard deviation (Wojniusz et al., 2016). Wojniusz et al. did report that emotional reactivity was possibly higher in girls treated with GnRHa although these results were not conclusive. Girls with early or precocious puberty are treated at a younger age so it is unclear to what extent these results apply to adolescents treated with GnRHa for gender dysphoria. Further studies are needed to assess if and what effects GnRHa have on various aspects of brain development in adolescence.

Opinions about the use of GnRHa vary (Vrouenraets, Fredriks, Hannema, Cohen-Kettenis, & de Vries, 2015). Arguments for the use of GnRHa that have been brought forward are the benefit of early treatment with GnRHa for mental health and quality of life (de Vries, Steensma, Doreleijers, & Cohen-Kettenis, 2011). Furthermore, it gives the adolescent and treatment team more time to explore the adolescent’s gender identity and treatment wishes (Hembree et al., 2017). If the adolescent pursues gender-affirming treatment, some surgeries may not be necessary or less invasive as secondary sex characteristics are less developed. Early treatment is correlated with better postsurgical outcomes, possibly because of a physical appearance more in line with the affirmed gender (Cohen-Kettenis & van Goozen, 1997; Leibowitz & de Vries, 2016). However, this may not be of equal importance to all adolescents and early puberty suppression also precludes certain surgeries such as penile inversion vaginoplasty by limiting penile growth. Some have argued that puberty-blocking treatment prevents devastating psychological and physical harms including suicide and that adolescents should therefore be able to access this treatment even without parental approval (Dembroff, 2019; Priest, 2019), but others have underscored that there is no evidence that puberty suppression prevents suicide and that the risk of suicide, although high, should not be overstated and should be seen in comparison with a clinical comparison group rather than the general population (Antommaria, Shapiro, & Conard, 2019; Baker, 2019; Zucker, 2019).

Arguments against the use of GnRHa that have been raised include possible long-term adverse effects on health, psychological, and sexual functioning (Laidlaw, Cretella, & Donovan, 2019; Richards, Maxwell, & McCune, 2019; Vrouenraets et al., 2015). Some state that adolescents may be unable to make far-reaching decisions at a young age, especially in the presence of comorbid psychiatric conditions, which are common among youth with gender dysphoria (Korte et al., 2008; Laidlaw et al., 2019; Vrouenraets et al., 2015). Furthermore, gender identity develops and may change during adolescence. Concerns have been raised that the use of GnRHa may influence this process and might increase the likelihood of persistence of gender dysphoria (Korte et al., 2008; Laidlaw et al., 2019; Richards et al., 2019; Stein, 2012; Vrouenraets et al., 2015). It is unknown if the use of GnRHa prevents resolution of gender dysphoria (Korte et al., 2008). Many prepubertal children with gender dysphoria no longer experience gender dysphoria in adolescence, and the experience of romantic and sexual attraction is thought to play an important role in this process (Steensma, Biemond, de Boer, & Cohen-Kettenis, 2011). Some may come to understand themselves as homosexual or bisexual (Steensma et al., 2011). GnRHa, by blocking sexual development, might interfere with this process (Korte et al., 2008). Another concern is that although GnRHa treatment is to be used as an extended diagnostic phase, the start of it may lead the adolescents and parents to assume that transgender outcome is the only possible outcome which may prevent exploration of other possibilities (Leibowitz & de Vries, 2016).

To gain more insight into the use of GnRHa in adolescents with gender dysphoria, the current study aims to document trajectories after the initiation of GnRHa, i.e., discontinuation of GnRHa, prolonged use of GnRHa, and initiation of gender-affirming hormones; to investigate the duration of GnRHa treatment; and to explore reasons for extended use and discontinuation of GnRHa.

## Method

### Participants

This is a single-center retrospective study. Out of 269 children and adolescents registered at the Curium-Leiden University Medical Centre gender clinic in Leiden, the Netherlands, 214 were pubertal and within the appropriate age range for treatment at our pediatric clinic. Out of these, 143 (67%) had started GnRHa treatment between November 2010 (when the clinic first started) and January 1, 2018. The study population consisted of these 143 adolescents (38 transgirls, 105 transboys). Not included in the study were children and adolescents in whom gender dysphoria was not diagnosed (*n* = 39), those who had coexisting problems that interfered with the diagnostic process and/or might interfere with successful treatment (*n* = 9), those that did not wish hormonal treatment (*n* = 4), those in whom the diagnostic evaluation was still ongoing (*n* = 10), and those who had stopped to attend appointments (*n* = 9).

Of adolescents who had started GnRHa, treatment status as of 1 July 2019 was reviewed. If they had used GnRHa monotherapy for more than 3 months longer than minimally required before the start of gender-affirming hormones according to the local protocol (see below for description of the treatment protocol), the reason for this was noted. The 3 months was chosen to select those who may have had a prolonged diagnostic phase rather than those in whom gender-affirming hormone therapy started slightly later due to logistical issues such as rescheduling of an appointment. Adolescents who had started GnRHa treatment and had stopped this treatment were included in a detailed review. Baseline characteristics such as age and gender and data on the start, duration, and discontinuation of treatment were recorded from the medical files, as well as reasons given for the discontinuation of GnRHa treatment and the adolescents’ and parents’ views on the treatment.

### Procedure

Before the start of GnRHa treatment, all adolescents had a diagnostic evaluation by a pediatric endocrinologist and mental health professional (MHP) to confirm the diagnosis of gender dysphoria according to the DSM-5 criteria (American Psychiatric Association, 2013), to assess the presence of any medical, psychiatric, or psychosocial problems that might interfere with treatment, to assess if the adolescent was able to give informed consent for the treatment and to confirm that puberty had started, as recommended by current guidelines (Hembree et al., 2017). This evaluation usually consisted of approximately six visits (more if necessary) of the adolescent with an MHP in 6–12 months in addition to interviews with parents/guardians. All adolescents gave written informed consent for the treatment. Informed consent from parents/guardians was also required if the adolescent was < 16 years old. After the start of GnRHa treatment, follow-up visits were scheduled with the pediatric endocrinologist and MHP, usually every 3 months in the first year and every 3–6 months thereafter, to evaluate satisfaction with the treatment, adequacy of puberty suppression, and any side effects. In the case of mental health issues (psychiatric morbidity but also issues such as difficulty to express oneself and doubts about one’s gender identity), adolescents were either seen more frequently by the psychologist of the gender team or referred to a local MHP for therapy.

According to the local protocol, adolescents were eligible for gender-affirming hormone treatment from the age of 16 years and after at least 6 months of GnRHa treatment. No maximum time of use of GnRHa was defined in the protocol. From 2016, adolescents who had already been treated with GnRHa for at least 3 years were eligible for gender-affirming hormone treatment from the age of 15 years. From 2017, those who had been treated with GnRHa for at least 2 years and were 15 years old were eligible. Before the start of gender-affirming hormones, evaluation by a MHP and pediatric endocrinologist took place to assess the indication, any contraindications, and ability to give informed consent for this treatment. If adolescents had discontinued GnRHa treatment, there was a follow-up appointment at which adolescents and parents were asked about current feelings regarding gender identity and how they looked back on the treatment.

## Results

**Fig. 1:**
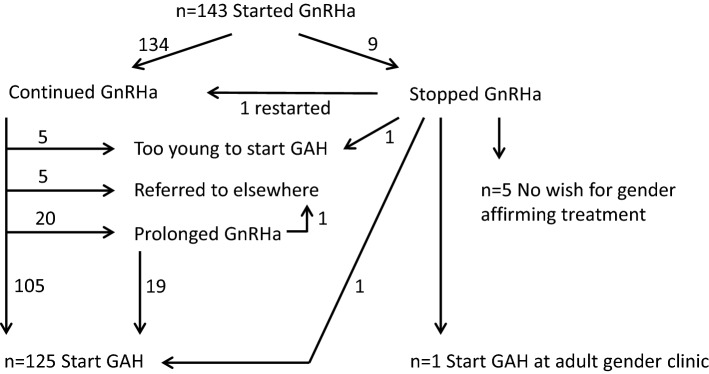
Flow chart showing the trajectories of adolescents who started GnRH analogue (GnRHa) treatment. *GAH* gender-affirming hormone treatment

During the study period, 143 adolescents started GnRHa treatment (38 transgirls, 105 transboys). Median age at the start of treatment was 15.0 years (range, 11.1–18.6 years) in transgirls and 16.1 years (range, 10.1–17.9 years) in transboys. Of these adolescents, 125 (87%, 36 transgirls, 89 transboys) subsequently started treatment with gender-affirming hormones after 1.0 (0.5–3.8) and 0.8 (0.3–3.7) years of GnRHa treatment (see Fig. 1). Median age at the start of gender-affirming hormones was 16.2 years (range, 14.5–18.6 years) in transgirls and 17.1 years (range, 14.9–18.8 years) in transboys. Five adolescents who used GnRHa had not started gender-affirming hormones at the time of data collection, because they were not yet eligible for this treatment due to their age. At the time of data collection, they had used GnRHa for a median duration of 2.1 years (1.6–2.8). Six adolescents had been referred to a gender clinic elsewhere for further treatment. One of these was 17 years old and eligible for gender-affirming hormones but initially indicated he needed more time to decide about testosterone treatment and subsequently stated that he wished to delay the start of this treatment until after his school examinations. The other five were not eligible yet due to their age at the time of referral. Nine had discontinued GnRHa treatment (see below), one of whom restarted GnRHa after 5 months. This individual and two others subsequently started gender-affirming hormone treatment (Fig. 1).

### Prolonged Use of GnRHa

Twenty adolescents (3 transgirls and 17 transboys) had used GnRHa for longer than minimally required by protocol. One was the transboy mentioned above who needed more time to decide about testosterone treatment. He had used GnRHa for 2.5 years when he was referred from the pediatric clinic to a clinic for adults elsewhere. The other 19 adolescents had subsequently started gender-affirming hormones. The median duration of GnRHa monotherapy in these 19 adolescents was 1.0 year (0.8–2.4). Reasons for prolonged use of GnRHa were (sometimes there was more than one reason): unstable situation due to family issues such as lack of parental support and/or acceptance of gender dysphoria (*n* = 6) or social problems such as lack of a safe home, excessive school absenteeism (*n* = 5); (psychiatric) comorbidity (*n* = 8) such as autism or depression; more time needed for decision about gender-affirming hormone treatment by the adolescent (*n* = 1) or for further diagnostics by the gender team (*n* = 1, because of non-binary aspects); and logistic issues such as missed/rescheduled appointments (*n* = 8; in 7 this was the only reason). The 11 adolescents who received prolonged GnRHa treatment because of mental health and/or psychosocial problems had regular (approximately monthly on average) appointments with a psychologist at the gender clinic (*n* = 5) and/or received support from a local MHP (*n* = 9) during this period.

### Discontinuation of GnRHa Treatment

From the 143 adolescents who started GnRHa treatment, nine (6%; one transgirl, eight transboys) stopped this treatment after a median duration of 0.8 years (0.1–3.0), at a median age of 15.0 years (13.4–18.9). Four individuals discontinued although they did wish further endocrine treatment because of gender dysphoria. One stopped treatment because of an increase in mood problems and suicidal thoughts and confusion attributed to GnRHa treatment and restarted treatment (gender-affirming hormone treatment) at an adult gender clinic elsewhere. He later indicated: “I was already fully matured when I started GnRHa, menstruations were already suppressed by contraceptives. For me, it had no added value” (transboy, age 19 years).

Another transboy experienced hot flushes, an increase in migraine, and had fear of injections in addition to stress due to problems at school and unrelated medical issues and therefore wished to temporarily discontinue GnRHa treatment after 4 months. He restarted 5 months later and subsequently started testosterone treatment. A third transboy experienced mood swings starting 4 months after he had begun GnRHa treatment. A year later, he started to frequently feel unwell and miss school. After 2.2 years, he developed severe nausea and rapid weight loss for which no cause was identified. Because of this deterioration of his general condition, he wished to discontinue GnRHa treatment after 2.4 years. He gradually recovered over the next 2 years. He subsequently started lynestrenol and testosterone treatment. The last adolescent had stopped GnRHa because his parents were unable to regularly collect medication from the pharmacy and take him to appointments for the injections. He subsequently started lynestrenol to suppress menses; he is not eligible yet for testosterone treatment.

The five others (3.5%) no longer wished gender-affirming treatment. One adolescent had been very distressed about breast development at the start of GnRHa. She later thought that she might want to live as a woman without breasts. She did not want to live as a boy and did not wish testosterone treatment and decided to discontinue GnRHa although she dreaded breast development and menstruation. Another adolescent had concurrent psychosocial problems interfering with the exploration of gender identity and did not currently wish treatment. When looking back on GnRHa treatment this individual said: “The decision to stop GnRHa to my mind was made by the gender team, because they did not think gender dysphoria was the right diagnosis. I do still feel like a man, but for me it is okay to be just me instead of a he or a she, so for now I do not want any further treatment” (adolescent assigned female sex at birth, age 16 years).

One adolescent felt more in between man and woman and therefore did not wish to continue treatment: “At the moment, I feel more like ‘I am’ instead of ‘I am a woman’ or ‘I am a man’” (adolescent assigned female sex at birth, age 16 years).

Another individual made a social transition while using GnRHa and shortly afterward decided to discontinue treatment. He indicated that he had fallen in love with a girl and had never had such feelings, which made him question his gender identity. At subsequent visits, he indicated that he was happy living as a man.

The last adolescent stated: “After using GnRHa for the first time, I could feel who I was without the female hormones, this gave me peace of mind to think about my future. It was an inner feeling that said I am a woman” (adolescent assigned female sex at birth, age 18 years).

The adolescents and parents were also asked about their views on GnRHa in the treatment protocol for gender dysphoria. All of them saw it as the first step in treatment, but it was also clear that it was used as an extended diagnostic phase. They all felt free to stop GnRHa. They had varying visions on the role of GnRHa in the treatment of gender dysphoria. Some stated it gave them time to think and feel who they were and what they wanted in the future and felt that without GnRHa treatment they would not have been able to make these decisions. Others stated that GnRHa should not be routinely offered before the start of gender-affirming hormones when adolescents are already fully matured, because of the lack of physical benefits. Instead, a consideration time of 6 months with psychological follow-up was suggested.

## Discussion

The great majority of adolescents who started GnRHa subsequently started gender-affirming hormones as soon as they were eligible for this treatment. Very few discontinued treatment, although slightly more than in previous studies in which cohorts of transgender adolescents were described. Out of 333 adolescents that had started puberty suppression at the VUmc gender clinic in the Netherlands up until December 2015, 1.9% stopped; reasons for discontinuation of GnRHa were not reported (Wiepjes et al., 2018). In the Canadian study by Khatchadourian, Amed, and Metzger (2014), one of 27 individuals who started GnRHa stopped the treatment due to emotional lability, not because the wish to pursue transition had subsided. In the current study, 6% of those who started GnRHa discontinued and 3.5% no longer wished gender-affirming treatment.

Several studies reviewed by Ristori and Steensma (2016) have found that much higher percentages (61–98%) of prepubertal children no longer experience gender dysphoria (“desist”) as adolescents. The period between 10 and 13 years seems to be a crucial period in which social changes (for example starting secondary school), the physical changes of puberty, and first romantic and sexual experiences may lead to either an increase or a decrease/resolution of gender dysphoria (Steensma et al., 2011). The adolescents that start GnRHa treatment have entered puberty and are mostly older than 13 years and may be past this critical period so that gender dysphoria may be more likely to persist. This may explain the lower percentage of resolution of gender dysphoria found in the studies of treated adolescents. In addition, the groups that started treatment in previous studies and in the current study consisted of selected adolescents that had had an extensive diagnostic process to establish if they met the eligibility criteria for treatment as well as the diagnostic criteria for gender dysphoria (Wiepjes et al., 2018). Alternatively, concerns have been raised that GnRHa treatment itself may increase the chances of persistence of gender dysphoria (Korte et al., 2008; Richards et al., 2019; Stein, 2012; Vrouenraets et al., 2015). Whether or not GnRHa treatment influenced gender identity development cannot be concluded from the current study due to its observational nature. The study does show that gender identity development was not suppressed in all, as a few adolescents discontinued GnRHa because they no longer experienced gender dysphoria, but it is unknown if gender dysphoria would have subsided in more adolescents in the absence of GnRHa treatment.

For one adolescent, the experience of falling in love made him doubt whether he was transgender. This is in line with previous findings that the first romantic experiences and the awareness of one’s sexual attraction play an important role in the resolution of gender dysphoria in adolescents (Steensma et al., 2011). This emphasizes the importance of this topic in the diagnostic evaluation. However, some adolescents may not have had any romantic or sexual experiences, especially if they present at an early age. In addition, transgender adolescents were shown to be less experienced, both sexually and romantically, compared to peers from the general population (Bungener, Steensma, Cohen-Kettenis, & de Vries, 2017). GnRHa treatment prevents the physical changes of puberty and is known to negatively affect sexual desire (Plosker & Brogden, 1994). Puberty suppression might thus decrease the chances of adolescents having romantic and sexual experiences which might in turn influence gender identity development (Korte et al., 2008). This was not true for the adolescent in the current study who fell in love while using GnRHa and then decided to discontinue treatment, but it is uncertain if more adolescents would have had such experiences if they had not used GnRHa.

Two individuals who discontinued GnRHa indicated that they did not feel either male or female. A non-binary gender identity appears to be becoming more common among adolescents presenting at gender clinics (Butler, De Graaf, Wren, & Carmichael, 2018). For these adolescents, it may be more difficult to find out and understand their own gender identity and it is unclear what constitutes optimal care for this group.

Experienced side effects played a role in the decision to discontinue GnRHa treatment in three adolescents. However, for none of the adolescents who stopped GnRHa in the current study, were potential long-term side effects a reason to decline or discontinue GnRHa treatment. Lack of information about long-term effects of GnRHa use was not considered an important problem by interviewed adolescents with gender dysphoria in the study by Vrouenraets, Fredriks, Hannema, Cohen-Kettenis, and de Vries (2016), but is seen as a major problem by many professionals (Vrouenraets et al., 2015).

In the current study, 13 adolescents who were eligible for gender-affirming hormone treatment used GnRHa monotherapy for longer than the minimum time required by protocol for reasons other than logistics. During this time, they received mental health support from a local MHP or from a psychologist from the gender team. This supports the idea that the time on GnRHa is used as an extended diagnostic phase where the adolescents can further explore their gender identity and treatment wishes and work on issues that might interfere with successful treatment. The great majority started gender-affirming hormones as soon as was possible within the treatment protocol, after a median duration of approximately one year. This does not mean that for them this time was not used as an extended diagnostic phase. Those who were youngest at the start of GnRHa were treated the longest, up to 3.8 years, with visits to the clinic every 3–6 months. In this period of growing up, becoming more independent, and discovering oneself, their development was followed by the team and discussed in relation to the treatment. Older adolescents, who presented after age 16 years, were often treated with GnRHa for the minimum period of 6 months. Generally, they were more mature than the younger adolescents at the start of the diagnostic process and many already had clear ideas about their treatment wishes. In adults, gender-affirming hormones are usually started directly after the diagnostic phase (Wiepjes et al., 2018).

The period of puberty suppression used in adolescents is considered worthwhile by some of the adolescents, as the individual in the current study who indicated it gave peace of mind to think about the future. On the other hand, some postpubertal adolescents perceived little benefit of the treatment, as stated by one transboy who discontinued GnRHa in the current study. A possible benefit of GnRHa treatment for fully matured transgender boys may be the suppression of menstrual bleeding. Alternative methods may be used to achieve this, although GnRHa are more effective than progestins to immediately and fully suppress menstruation (Tack et al., 2016). Furthermore, many adolescents do not wish to use continuous oral contraceptives because of the fact that they contain “female” hormones and because of fear that breast size may increase. Adolescents should be counseled on all available treatment options and their (side) effects so that they can make an informed choice.

The relatively small size of the cohort that was described is a limitation of the current study as well as its retrospective character. The duration of follow-up was limited, and in some of the adolescents who stopped GnRHa treatment because they no longer experienced gender dysphoria, gender dysphoria might recur later in life. The observational design does not allow conclusions about any possible effect of GnRHa treatment on gender identity development. A randomized controlled trial in adolescents presenting with gender dysphoria, comparing groups with and without GnRHa treatment, could theoretically shed light on the effect of GnRHa treatment on gender identity development. However, many would consider a trial where the control group is withheld treatment unethical, as the treatment has been used since the nineties and outcome studies although limited have been positive (de Vries et al., 2014; Smith, van Goozen, & Cohen-Kettenis, 2001). In addition, it is likely that adolescents will not want to participate in such a trial if this means they will not receive treatment that is available at other centers. Mul et al. (2001) experienced this problem and were unable to include a control group in their study on GnRHa treatment in adopted girls with early puberty because all that were randomized to the control group refused further participation. An alternative approach that has been suggested to gain more insight into the effect of treatment on gender identity development is to collect baseline data at the time of referral from adolescents who are on a long waiting list for diagnostic evaluation and treatment and compare the percentage of these adolescents in whom gender dysphoria is still present after a certain period of time to that in adolescents on GnRHa treatment (Zucker, 2019).

In conclusion, this study shows that a small number of adolescents discontinued GnRHa treatment because they no longer wished gender-affirming treatment. This indicates that not all adolescents and parents assume that transgender outcome is the only possible outcome and shows that gender identity can still fluctuate when using GnRHa, at least in some adolescents. However, gender dysphoria subsided in a small number of adolescents and it is uncertain if this would have been different without GnRHa treatment. Some adolescents used GnRHa for a prolonged period before starting gender-affirming hormones while regularly meeting with an MHP which is consistent with the use of GnRHa treatment as an extended diagnostic phase. The great majority who had started GnRHa treatment continued with gender-affirming hormones. It is important to take this into account when counseling adolescents who consider this treatment and their parents.
